# A Dual Biomarker Approach to Stress: Hair and Salivary Cortisol Measurement in Students via LC‐MS/MS

**DOI:** 10.1002/ansa.70003

**Published:** 2025-02-21

**Authors:** Muhammad K. Hakeem, Sundas Sallabi, Raghda Ahmed, Hana Hamdan, Amel Mameri, Mariam Alkaabi, Asmaa Alsereidi, Sampath K. Elangovan, Iltaf Shah

**Affiliations:** ^1^ Department of Chemistry College of Science United Arab Emirates University (UAEU) Al Ain UAE

**Keywords:** cortisol, hair cortisol, liquid chromatography‐tandem mass spectrometry (LC‐MS/MS), salivary cortisol, stress biomarker

## Abstract

Stress is a significant issue among students, affecting both their mental and physical health. In this study, we investigated cortisol levels, a key biomarker for stress, in students at the United Arab Emirates University (UAEU) during their exam period. Using a sensitive liquid chromatography‐tandem mass spectrometry (LC‐MS/MS) methodology we measured cortisol concentrations in hair and saliva samples and explored the potential correlation between exam‐induced stress and cortisol levels. The results revealed an increase in cortisol levels during the exam period, with male students showing an average hair cortisol concentration of 150.625 pg/mg and female students displaying an average of 77.756 pg/mg. Salivary cortisol levels ranged from 0.002 to 9.189 ng/mL, with an overall average of 4.505 ng/mL. Statistical analysis revealed significant differences in cortisol levels between male and female students, underscoring the impact of exam‐related stress on both acute and chronic stress markers. This study underscores the importance of addressing academic stress and suggests targeted strategies to mitigate its impact on student health ultimately fostering an environment encouraging both academic success and psychological well‐being within the student community. Future research directions include exploring additional clinical parameters and expanding the study population to further understand the long‐term effects of academic stress.

## 1 Introduction

Experiencing difficult or stressful events in life is associated with a deterioration in mental health [1]. This is particularly true for individuals who are exposed to such events throughout childhood and adolescence since these periods are known to raise the risk of depression and post‐traumatic stress disorder (PTSD) [2, 3, 4, 5]. Examining the expressions of stress in certain groups, including students whose specific challenges add to the complexity of stress experiences, becomes essential as we dig into the nuances of this condition. Given the unique pressures of academic life, understanding stress in students is essential. Academic pressures, social expectations and the quest for personal growth create an ambience where stress can profoundly impact cognitive functions and overall well‐being. This study aims to explore stress in university students during exam periods, specifically by measuring cortisol levels, a widely recognized stress biomarker.

Various methodologies, including physiological monitoring and biochemical signal analysis, have been used to study mental health, particularly in conditions such as depression and anxiety [6]. Monitoring biochemical biomarkers can help with understanding the neurobiological mechanisms underlying a number of mental illnesses, including clinical depression and bipolar disorder [7], as well as neurological diseases like Alzheimer's disease and dementia [8]. Monitoring certain biomarkers facilitates the early detection and diagnosis of mental health conditions and makes it easier to track the trajectory of the condition. Some methods may be used to estimate the stress brought on by exposure to unconventional situations, including assessments of the exposure to the circumstances and difficulty it causes, mental discomfort brought on by such experiences, and physiological reactions [9]. Changes in stress hormones like cortisol and adrenaline are frequently linked to psychological stress [10]. The main stress hormone, cortisol, is involved in controlling the stress response from the moment stress triggers until recovery from stressful events [11, 12, 13]. The well‐known hypothalamic–pituitary–adrenal (HPA) axis, one of the four primary neuroendocrine systems through which the hypothalamus and pituitary gland regulate neuroendocrine activity, produces the steroid hormone cortisol as its product [14]. In response to both long and short‐term stress, the HPA axis gets activated [15]. The adrenal gland constantly releases cortisol when the HPA axis is repetitively stimulated under long‐term stress [16, 17]. As a result, cortisol examination can serve as a useful tool for evaluating stress.

The body's cortisol levels are a useful indication of a number of vital processes and actions that are essential for maintaining homoeostasis and may also act as a biomarker in a number of illnesses [18]. Cortisol levels have especially been examined in relation to several ailments related to stress [10, 19, 20]. It is possible to extract cortisol from urine and blood. However, because sample timing, frequency, circadian rhythm and food might alter the results, it can be challenging to assess chronic stress using plasma or urine [21, 22, 23, 24]. There is a need for frequent sampling and specific storage requirements (such as refrigeration or freezing), potentially augmenting the workload on researchers while potentially reducing participant adherence. The cortisol measurement from blood or urine provides insight into the extent of short‐term stress. Furthermore, blood collection is an invasive procedure that might stress patients out and raise their cortisol levels. Therefore, a suitable cortisol testing sample is required.

Since there is a strong correlation between salivary cortisol levels and unbound cortisol levels in serum, salivary cortisol is thought to reflect changes in free plasma cortisol, making measuring cortisol levels in saliva a reliable alternative [25, 26]. Saliva, a bodily fluid with demonstrated diagnostic potential [27], can reflect cortisol levels influenced by stress. Because saliva does not coagulate and is stable for 24 h at room temperature and for a week at 4°C, it is the optimal biological fluid for diagnostic purposes. Saliva is being used in medical diagnostics since it is affordable, non‐invasive, painless, and practical. Saliva is made up of a variety of chemicals, the concentration of which may be utilized to identify early pathogenic changes quickly and easily in people [28, 29]. Using precise and sensitive immunological or biochemical techniques, saliva components may be inexpensively identified [30]. Saliva is not the perfect biological material despite the benefits we have outlined above. Numerous variables, including age, gender, drugs, menstrual cycle, pregnancy, caffeine intake and alcohol usage, might affect each person's salivary composition [31].

In addition to measuring cortisol levels in blood plasma, saliva and urine samples, cortisol can also be assessed from human hair [32, 33]. The hair cortisol concentration (HCC) appears to be a promising new way of assessing chronic stress [34]. Through passive diffusion from the circulation, cortisol penetrates hair at the hair stem medulla [35]. However, other research found that the hair follicle contains an HPA‐like axis, allowing it to synthesize cortisol [36]. The hair samples taken from within 1 cm of the scalp provide insights into the cortisol release that occurred in the past month, as hair grows approximately 1 cm each month [37]. Similarly, when a 3 cm hair sample is collected from the nearest scalp region, it can assess chronic stress levels experienced during the preceding three months [38]. The advantages of using a hair sample over other sampling techniques are numerous. For instance, it may be kept at room temperature, and acute stress and dietary intake have no effect on HCC [22]. Hair sampling is furthermore non‐invasive [39]. Hair cortisol levels have been proposed in this context as a retrospective, long‐term biomarker of HPA axis functioning, associated with stress, emotional and behavioural symptoms [40, 41], and correlation with the level of cortisol in saliva.

Enzyme‐linked immunosorbent assay (ELISA) [42], CORT assay [43], liquid chromatography‐tandem mass spectrometry (LC‐MS/MS) [44, 45, 46], HPLC‐Fluorescence [47] and chemiluminescence analysis (CLIA), electrochemical detection [48, 49], radio immunoassay (RIA) [50], and immunosensors [51, 52, 53] are different techniques for measuring cortisol level in hair and saliva [15]. Different methods of evaluation yielded various relationships between cortisol concentration and stress over time. The most widely used techniques for assessing cortisol in ordinary clinical procedures are cortisol immunoassays, which rely on antibodies that recognize cortisol. Radioimmunoassay, ELISAs, chemiluminescent immunoassay, electro‐chemiluminescent immunoassay and fluorescence immunoassay are included in cortisol immunoassays [18, 54]. As a quick and reliable way for evaluating cortisol status, immunoassays are typically the method of choice. However, changes in albumin levels may have an impact on the immunochemical estimation of total cortisol in plasma samples [55, 56]. In addition, immunoassays are frequently used to measure salivary cortisol because they may offer the low detection limits necessary to quantify the hormone present in salivary samples, even at the lowest point in the daily rhythm [55]. Although cortisol immunoassays are extensively employed, their analytical specificity can be limited due to antibodies that exhibit cross‐reactivity with other steroids present in the sample, and there is also a possibility of preanalytical disruptions occurring [57]. The great specificity of LC‐MS/MS procedures, on the other hand, makes them more popular in clinical research facilities, particularly when interferences are thought to be present. In addition, the LC‐MS/MS technology is regarded as the accepted standard for HCC analysis [58].

This study presents a novel approach by combining the assessment of both salivary and hair cortisol levels to provide a comprehensive understanding of stress among university students. Previous research has predominantly focused on short‐term cortisol fluctuations in saliva, which reflect immediate stress responses. However, our study expands on this by also measuring hair cortisol, which provides a retrospective, long‐term view of chronic stress over several months. This dual approach offers deeper insights into both immediate and chronic stress responses, making it particularly relevant for understanding the complexities of student stress during critical academic periods. Furthermore, the application of LC‐MS/MS in our analysis enhances the specificity and accuracy of cortisol detection compared to conventional immunoassays, which can suffer from cross‐reactivity and pre‐analytical disruptions. This methodological choice underscores the rigorous nature of our research, positioning it at the forefront of stress‐related studies in academic settings.

## 2 Materials and Methods

### 2.1 Standards and Reagents

Hydrocortisone and hydrocortisone‐D4 (used as the internal standard) were acquired from Labco Ltd. in Dubai, UAE. The chemicals dichloromethane, methanol, formic acid, as well as LC‐MS grade water and acetonitrile, were obtained from Emirates Scientific and Technical Supplies Ltd, also located in Dubai, UAE.

### 2.2 Sample Collection

In adherence to rigorous ethical standards and with approval from the UAE University Ethics Committee (UAEU Ref# SNA/fa/19‐15), our study engaged a diverse group of students from UAE University, encompassing both males and females. The participants, demonstrating a commitment to the research endeavour, provided informed consent by duly signing the required forms. The collection process was conducted with precision, ensuring the ethical and respectful treatment of participants. The sample collection took place twice in the academic year. The baseline hair samples were collected 1 month after the start of the academic year and follow‐up samples were collected right after the exam period. Hair samples were obtained from a cohort consisting of 60 male and 37 female volunteer students. These samples were obtained from the posterior vertex, a 3 cm segment near the scalp, aiming to capture cortisol levels over the past 3 months. Similarly, saliva samples were also collected twice from the volunteer students. The baseline samples were collected at the start of the academic year and follow‐up samples were collected right after the exam. Saliva samples were also collected from the same volunteer students. Before saliva collection, participants were instructed to gargle with water and collect saliva with a 5.000 mL plastic tube after gargling. Unlike hair samples, saliva samples represent cortisol levels in the immediate stress state of the participants and not throughout a period. All the samples were collected at the end of final exams. All collected samples were subjected to a systematic labelling protocol. Each sample was individually sealed in appropriately designated plastic bags, safeguarding the integrity of the specimens throughout the storage and analytical phases of the study. The diverse cohort strengthens the study's findings, offering insights into cortisol dynamics in university students during a period of heightened stress.

### 2.3 Cortisol Extraction From Saliva Samples

The saliva samples were collected from the volunteer students and processed for cortisol extraction using the protein precipitation technique. Acetonitrile was added to the samples to precipitate the protein and the mixture was kept at 4°C for 5 min until centrifugation. The samples were centrifuged at 1500 rpm for 10 min using a Beckman TJ‐6 centrifuge (Beckman, High Wycombe, UK) and the supernatant was transferred to another tube for the concentration of sample using nitrogen gas sample concentrator (Techne, Bibby Scientific, Vernon Hills, IL, USA). The dried samples were then reconstituted by adding 100 µL of mobile phase (10% water with 0.1% formic acid [Mobile Phase A] and 90% methanol with 0.1% formic acid [Mobile Phase B]) for LC‐MS/MS analysis. This extraction method ensures minimal protein interference and maximizes cortisol recovery from saliva, allowing for accurate quantification.

### 2.4 Extraction of Analyte From Hair Samples

The hair samples underwent a thorough cleansing procedure using dichloromethane to remove any impurities, dyes, or external substances adhering to the strands. Following that, the hair was immersed in dichloromethane for a period of 5 min each. Afterwards, the hair samples were left to dry in ambient air conditions for approximately 15 min. After being dried, the hair samples were pulverized by utilizing a Mini‐ball mill (Pulverisette 23, Fritsch, Idar‐Oberstein, Germany) for a duration of 15 min at a frequency of 50 oscillations per second. Later, a precise measurement of 20.000 mg of finely ground hair was conducted, and 50.000 µL of hydrocortisone‐D4 with a concentration of 1.000 ng/mL was added to all hair samples, excluding the blank matrix samples. To facilitate further processing, 2.000 mL of methanol was added to the hair samples, which were then subjected to 1 h of sonication in an ultrasonic bath (Branson 5800, Branson, Danbury, CT, USA) maintained at a temperature of 40°C. Subsequently, the samples were vortexed for a duration of 2 min. Following the ultrasonication, the resulting mixture was centrifuged (Beckman TJ‐6 centrifuge, High Wycombe, UK) at 2000 × *g* for 20 min. After centrifugation, the upper organic layer was carefully separated into new Pyrex glass test tubes using Pasteur pipettes. Then, the clear extract was evaporated at a temperature of 40°C using a nitrogen gas sample concentrator (Techne, Bibby Scientific, Vernon Hills, IL, USA). To maintain the integrity of the peak shape and prevent any potential retention time drift, the dried extract was effectively restored by incorporating 100.000 µL of a methanol‐water solution (in a 20:80 ratio).

### 2.5 LC‐MS/MS Analysis

In this study, cortisol levels in both hair and saliva samples were quantified using a highly sensitive and specific LC‐MS/MS method. The system consisted of a tandem mass spectrometer paired with a UHPLC system, utilizing gradient elution to optimize analyte separation. The LC‐MS/MS operated in Multiple Reaction Monitoring (MRM) mode, utilizing the formate adduct [M+HCOO^−^] of the target analyte as precursors. For the mobile phase, solvents of LC‐MS grade were selected to ensure the purity and reliability of the analytical procedure. Two mobile phases, Mobile Phase A consisting of water with 0.1% formic acid and Mobile Phase B consisting of methanol with 0.1% formic acid, were prepared to the highest standards. A gradient method was employed to optimize the separation process for our targeted analytes. The specific gradient applied during the experiment is shown in Figure S1. This gradient elution strategy allows for the effective separation of cortisol, optimizing the sensitivity and specificity of the LC‐MS/MS analysis. Further details regarding the LC‐MS/MS methodology and the associated validation results are provided in the Supporting Information.

### 2.6 Method Validation

A validated LC‐MS/MS method was employed to analyse cortisol and its isotopic analogue Cortisol D4 in both hair and saliva samples. The assay's precision, accuracy, linearity, specificity and recovery were confirmed following the standards set by the US Food and Drug Administration (FDA) [59]. The optimized MRM parameters were used to enhance analytical accuracy. For a detailed description of the assay parameters, including MRM transitions, collision energies and ionization modes, as well as the comprehensive validation results including the recovery rates, precision, accuracy and detection limits, please refer to the Supporting Information.

This study introduces an innovative approach by employing LC‐MS/MS to quantify cortisol levels in both saliva and hair samples from male and female students. This dual‐sample analysis represents an analytical breakthrough, providing a comprehensive profile of cortisol fluctuations associated with stress. Unlike traditional methods that often rely on a single type of sample, this approach allows for the assessment of both acute (via saliva) and chronic (via hair) stress levels, offering a more nuanced understanding of stress impacts. The ability to accurately quantify cortisol in hair samples is particularly noteworthy, as it provides a non‐invasive, long‐term indicator of stress exposure. This represents a significant advancement over existing methods that may rely on more invasive procedures or only provide a snapshot of stress levels.

## 3 Results and Discussion

### 3.1 Validation Results

A comprehensive validation was performed for the analytical method used in this study to detect and quantify cortisol levels in student samples. Hair samples were collected from UAEU student volunteers, with cortisol concentrations measured in the 3 cm segment closer to the scalp in each sample. These concentrations are supposed to represent cortisol levels throughout the past 3 months. In contrast, saliva samples were collected to assess acute cortisol levels, reflecting the participant's current physiological state at the time of collection. This dual‐sample approach enables a comprehensive analysis of both long‐term and short‐term stress responses. The LOQ values for cortisol analysis were determined to be 15.000 pg/mg for hair samples and 0.450 ng/mL for saliva samples, as established during method validation (Tables S2 and S3). Importantly, all analysed samples exceeded the respective LOQ values, ensuring the accuracy and reliability of the cortisol measurements. All samples were gathered during the academic year to investigate stress levels during a high‐pressure academic setting. The identification and quantification of cortisol were achieved by analysing its retention time and the relative abundance of its corresponding product ions. The chromatographic conditions facilitated the precise separation, identification and quantitation of cortisol, confirming the specificity and accuracy of the methodology. The results underscore the method's robustness, with detailed validation outcomes, including precision, accuracy and representative chromatograms, available in the Supporting Information.

### 3.2 Sample Analysis

**FIGURE 1:**
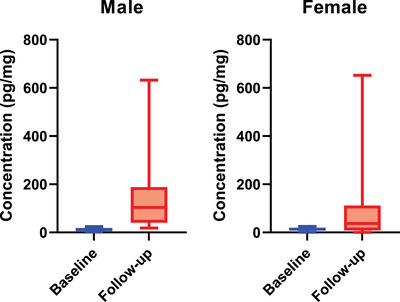
Distribution of hair cortisol concentrations among baseline and follow‐up samples of male (*N* = 60) and female (*N* = 37) students.

Hair and saliva samples were collected from volunteer students and cortisol concentrations were measured. The hair samples were collected at the beginning and end of the semester, after the final exam period with the cortisol levels intended to represent the students' stress levels during the period. Cortisol was detected in the majority of the female participants, many of whom are either taking anti‐depressants or have some other health issues. In comparison, the rest are healthy, and their cortisol levels are assumed to correlate with stress (Figure S3). The mean cortisol level of hair in female students right after final exams was 77.756 pg/mg. For male students, cortisol was detected in most participants, with an average of 150.625 pg/mg in post‐exam (follow‐up) samples, indicating significant stress in this group. Figure 1 illustrates box and whisker plots depicting cortisol concentrations in hair samples from both male and female students for both baseline and follow‐up samples. These plots visually convey the distribution of the data, including medians, quartiles and outliers. The broad range of values and the presence of outliers indicate the diverse chronic stress responses among students. Typical HCCs in healthy individuals range from 1 to 25 pg/mg, with elevated levels often associated with chronic stress conditions [34, 60, 61]. Our findings of 15–652 pg/mg suggest a substantial stress burden among students, likely intensified due to exam pressures.

Another potential factor influencing hair cortisol levels is the presence of cortisol in sebum. Sebum, secreted from the skin's sebaceous glands, may act as a medium for cortisol transport from pores onto the hair shaft. This mechanism could contribute to the cortisol detected in hair samples, especially in regions with high sebaceous activity. While this pathway has not been explored in detail in this study, it underscores the need for future investigations to quantify the extent of sebum‐mediated cortisol deposition and its implications for hair cortisol analysis. Variability in hair cortisol levels may also arise from differences in the strength of cortisol binding to the keratin matrix, which could be influenced by hair colour. For example, darker hair may bind cortisol more strongly due to higher melanin content, potentially leading to higher cortisol concentrations compared to lighter hair. In addition, individual differences in cortisol production, metabolism and excretion related to ethnicity may contribute to variability in measured cortisol levels. While our study did not specifically address these factors, future research should explore their potential impact on hair cortisol analysis. Such investigations would provide a more nuanced understanding of how biological and physiological variations influence cortisol deposition in hair.

**FIGURE 2:**
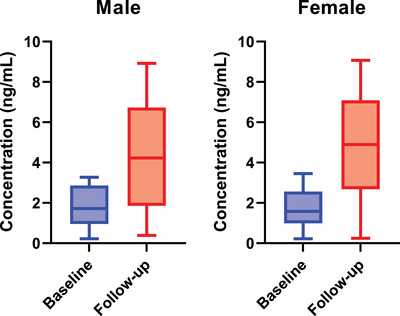
The plot showing the distribution of salivary cortisol concentrations among baseline and follow‐up male (*N* = 60) and female (*N* = 37) students.

Saliva samples from the same students were collected for analysis just before their exam to assess acute stress responses. Unlike hair samples, which reflect long‐term stress, saliva samples represent cortisol levels in the participant's current state of stress. Figure 2 shows box and whisker plots representing the cortisol levels in saliva samples of both baseline and follow‐up male and female students. Salivary cortisol levels ranged from 0.002 to 9.189 ng/mL, with an average of 4.402 ng/mL for follow‐up male and 4.672 ng/mL for follow‐up female samples (Figure S4). Figure 2 provides an overview of the data distribution, highlighting medians, quartiles and potential outliers. The broad range of values, as well as the presence of outliers, reflects significant variability in acute stress responses in the student population. Typical salivary cortisol levels in non‐stressed individuals range from 0.2 to 3.5 ng/mL, with acute stress potentially increasing these values [62, 63]. Our findings of 0.002 to 9.189 ng/mL indicate that students experienced a wide spectrum of stress levels, with some values far exceeding typical stress responses, likely due to the high‐pressure exam environment.

The results of this study reveal a strong association between exam‐related stress and elevated cortisol levels in UAEU students. The analysis of both hair and saliva samples provides a comprehensive view of both chronic and acute stress responses. We have noticed that male students exhibited higher average levels of hair cortisol than females, suggesting potential differences in chronic stress responses between genders. This difference may reflect not only physiological stress responses to exams but also broader societal or cultural pressures. In some settings, males may face increased expectations to perform academically or professionally, which could contribute to higher baseline and exam‐related stress levels. Future studies should explore how social and cultural factors influence chronic stress biomarkers to better contextualize these findings. However, the variability observed in female salivary cortisol levels indicates diverse acute stress responses, possibly due to individual differences in coping mechanisms or stress perception. While this study detected cortisol in the majority of female participants, some of whom were managing health issues or were taking antidepressants, the precise impact of these factors on cortisol levels was not systematically assessed. Future studies should aim to collect detailed participant health data to evaluate the extent to which such factors affect cortisol measurements and stress‐related conclusions. This will enhance the robustness of future findings. The findings highlight the need for personalized stress management strategies. Given the broad range of stress responses among students, these strategies should consider individual physiological and psychological differences. Furthermore, this study highlights the potential of using LC‐MS/MS techniques to accurately quantify both acute and chronic stress biomarkers, offering an important tool for future research into stress and health.

### 3.3 Statistical Analysis

A paired *t*‐test was conducted to compare cortisol levels in both hair and saliva samples of male and female students at two‐time points: baseline (before exams) and follow‐up (after exams). The results showed significant increases in cortisol levels across both sample types and genders, indicating that stress levels rose considerably during the exam period. For salivary cortisol, male students exhibited a highly significant increase (*p* < 0.0001) with a mean difference of 2.602 ng/mL between baseline and follow‐up samples, while female students also showed a significant increase (*p* < 0.0001) with a mean difference of 2.950 ng/mL. Similarly, hair cortisol levels showed a significant increase in both male (*p* < 0.0001, mean difference = 138.4 pg/mg) and female students (*p* = 0.0019, mean difference = 64.82 pg/mg). Overall, the statistical analysis demonstrated that the elevated cortisol levels both acute and chronic are statistically significant, with both exceeding the normal physiological range. This confirms that the elevated cortisol levels are not a result of random variation but are indicative of exam‐related stress among the student population. In addition to the paired t‐test analysis, estimation plots were generated to visualize the mean differences between baseline and follow‐up cortisol levels in both hair and saliva samples for male and female students (Figures S5 and S6). These plots provide a clear representation of the magnitude of the differences and their associated confidence intervals, offering a more intuitive understanding of the data. The plots highlight the mean of differences, which further confirms the significance of the changes observed between baseline and follow‐up samples.

A correlation analysis was also performed between saliva cortisol (acute stress marker) and hair cortisol (chronic stress marker) levels to investigate the relationship between acute and chronic stress. Scatter plots of saliva versus HCCs were generated for baseline and follow‐up samples (Figure S7). The analysis revealed a weak and statistically non‐significant correlation at baseline (*r* = 0.25, *p* > 0.05). However, at follow‐up, a significant positive correlation was observed (*r* = 0.68, *p* < 0.001). These findings suggest that while baseline samples demonstrated a weak and statistically non‐significant correlation between acute and chronic stress markers, the follow‐up samples exhibited a much stronger positive correlation. This temporal shift may be attributed to physiological adaptations to prolonged stress exposure during the semester, as the body's acute and chronic stress responses become more synchronized. These findings provide insights into how stress manifests over time, emphasizing the importance of longitudinal studies in stress biomarker research.

## 4 Conclusions

This study provides a comprehensive analysis of cortisol levels in students, demonstrating a significant relationship between academic stress and physiological responses. By measuring cortisol concentrations in both baseline and follow‐up hair and saliva samples, we captured the dual aspect of chronic and acute stress. The observed variations in cortisol levels between hair and saliva samples as well as between genders offer valuable insights into the complex dynamics of stress. The application of both hair and saliva cortisol analysis, supported by robust statistical findings (*p* < 0.0001), further strengthens the study's conclusions. The potential negative impact of prolonged high cortisol levels on human health and well‐being highlights the importance of addressing exam‐related stress in the educational field. The study suggests that necessary efforts should be taken to reduce hazardous exam‐related stress among students. Continued efforts in this direction can contribute to both the mental well‐being and academic success of students facing the challenges of stress and depression.

### 4.1 Strategies for Stress Reduction

Numerous strategies can be strategically implemented to alleviate exam‐related stress among students. Organizational tools, such as calendars, can be instrumental in helping students manage their time effectively. Regular study breaks, coupled with an emphasis on physical and mental well‐being through activities like exercise, balanced nutrition and adequate sleep, contribute significantly to stress reduction. Teaching others, creating portable study spaces and varying environments, and establishing study routines tailored to individual needs are additional effective strategies. Encouraging healthy habits, such as avoiding all‐night study sessions, and promoting laughter and communication with peers, teachers or therapists, collectively form a comprehensive approach to mitigating stress.

### 4.2 Future Directions

Future studies will focus on further elucidating the implications of the observed cortisol fluctuations by incorporating additional clinical parameters such as heart rate variability (HRV), blood pressure and immune markers like cytokines. HRV, a known marker of autonomic nervous system activity, could offer additional insights into the physiological impact of prolonged stress. Similarly, examining changes in blood pressure and immune function in relation to cortisol levels could highlight the broader health implications of stress, including susceptibility to illness and chronic disease. Further investigation into exam‐related stress using more significant, more diverse populations and long‐term monitoring of cortisol levels could reveal the lasting effects of stress on students' well‐being. In addition, employing segmental analysis of hair cortisol to analyse specific periods of heightened stress would provide more detailed insights into the relationship between cortisol levels and stress exposure.

## Author Contributions

**Muhammad K. Hakeem**: methodology and investigation, writing original draft, writing, review, & editing. **Sundas Sallabi**: methodology and investigation, writing original draft, writing, review & editing. **Raghda Ahmed**: methodology and investigation, writing, review & editing. **Hana Hamdan**: methodology and investigation, writing, review & editing. **Amel Mameri**: methodology and investigation, writing, review & editing. **Mariam Alkaabi**: methodology and investigation, writing, review & editing. **Asmaa Alsereidi**: methodology and investigation, writing, review & editing. **Sampath K. Elangovan**: methodology and investigation, writing, review & editing. **Iltaf Shah**: conceptualization, funding acquisition, supervision, resources & project administration, writing, review & editing.

## Ethics Statement

The study was conducted in accordance with the Declaration of Helsinki and approved by the Ethics Committee of United Arab Emirates University (protocol code: SNA/fa/19‐15).

## Consent

Informed consent was obtained from all students involved in the study.

## Conflicts of Interest

The authors declare no conflicts of interest.

## Supporting information



Supporting Information.

## Data Availability

The data that support the findings of this study are available from the corresponding author upon reasonable request.
