# Supplementary material for: A Dual Biomarker Approach to Stress: Hair and Salivary Cortisol Measurement in Students via LC‐MS/MS
Source: Anal Sci Adv. 2025 Feb 21;6(1):e70003. doi: 10.1002/ansa.70003 (PMC11845309; doi:10.1002/ansa.70003)

Supplementary Information

A Dual Biomarker Approach to Stress: Hair and Salivary Cortisol Measurement in Students via LC-MS/MS

Muhammad K. Hakeem, Sundas Sallabi, Raghda Ahmed, Hana Hamdan, Amel Mameri, Mariam Alkaabi, Asmaa Alsereidi, Sampath K. Elangovan ^1^, Iltaf Shah*

Department of Chemistry, College of Science, United Arab Emirates University (UAEU), Al Ain P.O. Box 15551, Unite Arab Emirates

***** Correspondence: altafshah@uaeu.ac.ae

**Instrumentation and Chromatographic Conditions**

The LC-MS/MS system incorporates an 8060 tandem mass spectrometer, paired with the Nexera ultra-high-pressure liquid chromatography (UHPLC) system from Shimadzu, Japan. The Nexera X2 UHPLC is composed of various modules, including a pump, auto-sampler, column oven, and degasser. To achieve chromatographic separation, an Ascentis Express F5 column with dimensions 150mm × 2.1mm × 2.7 μm was utilized. Additionally, an HPLC guard column was connected to the column for physical filtration. The choice of these components, coupled with the utilization of small particle columns, significantly contributed to the precision and efficiency of the separation process.

**Mass Spectrometry and Detection**

The LC-MS/MS system operated in Multiple Reaction Monitoring (MRM) mode, utilizing the formate adduct [M+HCOO–] of the target analyte as precursors. This mode of operation ensured the acquisition of accurate and reliable data throughout the analysis. Maintaining a consistent temperature of 40°C within the column compartment and a mobile phase flow rate of 0.300 mL/min, we prioritized the stability and precision of our analytical conditions. The auto-sampler's capabilities, including high-speed injections, utilization of multi-solvent loops, and injection port rinsing, further enhanced the efficiency of the overall analytical process. For instrument control, data handling, and analysis, the LCMS-8060 employs Shimadzu's Lab Solutions software.

**LC-MS/MS Assay Description**

The **figure S1** showcases the dynamic changes in the composition of mobile Phase A and B during analysis. At the initial time point, the system begins with 100% mobile Phase A, ensuring the exclusive presence of the aqueous component. Between 1 and 4.9 minutes, a gradual transition takes place, with mobile Phase B gradually increasing from 0% to 100%. This shift introduces the organic component to the mobile phase, initiating the elution of cortisol. This organic-rich environment continues until 9.9 minutes, facilitating the complete elution of cortisol from the chromatographic column. Between 9.9 and 10 minutes, the system transitions back to the initial conditions, with mobile Phase A regaining its dominance. This marks the end of the cortisol elution phase. The elution profile stabilizes and maintains the initial conditions from 10 to 12 minutes, preparing the system for subsequent analyses.

**Figure S1:** Mobile phase gradient elution profile during LC-MS/MS analysis

Method Validation

A novel approach was devised and verified, adhering to the method validation standards set forth by the US Food and Drug Administration (FDA). The LC-MS/MS analysis encompassed a comprehensive evaluation of precision, accuracy, linearity, specificity, and recovery. The optimized Multiple Reaction Monitoring (MRM) parameters were employed to facilitate the analysis of cortisol and its isotopic analog Cortisol D4 in both hair and saliva samples. These parameters, as outlined in **Table S1**, define the essential specifications for tracking the analyte during the experiment. These parameters, as outlined in **Table S1**, define the essential specifications for tracking the analyte during the experiment. For cortisol, the protonated molecule was observed at m/z 407.25, with three corresponding product ions at m/z 331.15, 297.30, and 282.20. The MRM transitions were carefully monitored with a dwell time of 100 milliseconds, ensuring precise data acquisition. Similarly, for Cortisol D4, the precursor ion was observed at m/z 411.20, with corresponding product ions at m/z 335.20, 301.15, and 286.25. Collision energy settings (CE) for each transition were optimized **(Table S1)**. Additionally, pre-bias voltages (Q1 pre-bias and Q3 pre-bias) were meticulously applied for stable ionization and transmission during ionization mode. Negative ionization mode was employed for both cortisol and Cortisol D4 **(Table S1)**. This rigorous optimization process ensures the reliability and accuracy of the LC/MS/MS analysis, laying a robust foundation for the subsequent quantification of cortisol levels in the studied samples.

**Table S1.** MRM transitions of Cortisol and Cortisol D4.

| **Compounds** | **Precursor (Q1) (m/z)** | **Product (Q3) (m/z)** | **Dwell Time (msec)** | **CE (eV)** | **Q1 pre Bias (V)** | **Q3 pre Bias (V)** | **Ionization** |
| --- | --- | --- | --- | --- | --- | --- | --- |
| **Cortisol** | 407.25 | 331.15 | 100 | 15 | 20 | 20 | Negative |
|  | 407.25 | 297.30 | 100 | 32 | 20 | 13 |  |
|  | 407.25 | 282.20 | 100 | 39 | 21 | 12 |  |
| **Cortisol D4** | 411.20 | 335.20 | 100 | 16 | 20 | 20 | Negative |
|  | 411.20 | 301.15 | 100 | 33 | 12 | 13 |  |
|  | 411.20 | 286.25 | 100 | 39 | 21 | 12 |  |

Analysis of the quality controls (QCs) at three distinct concentrations; low (LQC), medium (MQC), and high (HQC) was used to determine the linearity, intra and inter-day precision, and accuracy. The clean quality controls at the three concentrations were gently dried down with a spray of N_2_ before being reconstituted for the recovery experiment. The % Accuracy was calculated by the given formula:

$\% Accuracy=\left( \frac{Mean Value}{Nominal Value} \right)\times100$ (1)

The Inter/Intra -day precision was calculated using following equation after obtaining data from the analysis of quality control samples.

$\% CV=\left( \frac{Standard Deviation}{\mathrm{Mean}} \right)\times100$ (2)

The following equation was used for calculation of % recovery.

$\% Recovery=\left( \frac{Mean extracted QC Values}{Mean unextracted QC Values} \right)\times100$ (3)

To determine the lower limit of detection (LOD), we established it by comparing the signal-to-noise ratio (S/N) of the instrument to the lowest concentration of the analytes. The lowest concentrations of the analytes were determined by gradually reducing their concentrations until an LC-MS/MS detector response, which was three times the level of the instrument's background noise, was observed. To assess the analyte recovery, quality control (QC) samples were prepared in methanol. These samples were subsequently dried, reconstituted in the mobile phase, and injected into the LC-MS/MS system. The area under the curve for all QC samples was calculated. Subsequently, QC samples with the same concentrations spiked to blank samples, and the mixture was subjected to the normal extraction protocol. After that, the samples were dried, reconstituted, and injected into the LC-MS/MS system, and the area under the curve was calculated for these samples as well. Intra and inter-assay accuracy and precision were calculated based on quality control samples analyzed for hair and saliva samples **(Table S2 & S3)**. Average recovery for the hair samples was found to be 75.330 % while that for the saliva sample was 86.050 % as shown in **table S2 & S3** respectively. During the validation study, it was observed that the method displayed favorable sensitivity, specificity, and linearity. Furthermore, it exhibited a sensitive limit of quantitation and limit of detection for all the analytes, as depicted in **table S2 & S3.**

**Table S2.** Intraday and Interday precision and accuracy for hair samples.

| **Quality control** | **Concentration (pg/mg)** | ***Intraday*** | | ***Interday*** | | **r^2^** | **LOD (pg/mg)** | **% Recovery** | **Linearity Range (pg/mg)** |
| --- | --- | --- | --- | --- | --- | --- | --- | --- | --- |
|  |  | **Accuracy %** | **Precision (% CV)** | **Accuracy %** | **Precision (% CV)** |  |  |  |  |
| LQC | 62.500 | 110.100 | 3.050 | 107.300 | 5.400 | 0.998 | 5.000 | 75.330 | 15.000-2000.000 |
| MQC | 1000.000 | 110.800 | 1.700 | 108.700 | 7.910 |  |  |  |  |
| HQC | 1500.000 | 103.500 | 1.520 | 103.940 | 4.900 |  |  |  |  |

**Table S3.** Intraday and Interday precision and accuracy for saliva samples.

| **Quality control** | **Concentration (ng/mL)** | ***Intraday*** | | ***Interday*** | | **r^2^** | **LOD (ng/mL)** | **% Recovery** | **Linearity Range (ng/mL)** |
| --- | --- | --- | --- | --- | --- | --- | --- | --- | --- |
|  |  | **Accuracy %** | **Precision (% CV)** | **Accuracy %** | **Precision (% CV)** |  |  |  |  |
| LQC | 0.714 | 110.180 | 2.550 | 106.750 | 2.010 | 0.997 | 0.156 | 86.050 | 0.450-25.000 |
| MQC | 2.850 | 97.420 | 1.140 | 95.820 | 1.500 |  |  |  |  |
| HQC | 11.430 | 108.740 | 2.010 | 106.580 | 1.720 |  |  |  |  |

LOD: Limit of detection, r2: Correlation coefficient, % CV: Coefficient of variation

**Validation Results**

The validation of the LC-MS/MS method demonstrated high sensitivity and specificity for cortisol detection in both hair and saliva samples. The chromatographic conditions enabled effective separation, and the MRM mode provided accurate quantitation. Representative chromatograms for spiked blank hair and saliva samples, along with internal standards, are provided in **Figures S2 A and S2 B**. The chromatograms highlight distinct and clearly defined peaks at their specific retention times for all the analytes, including hydrocortisone and hydrocortisone-D4.

**Figure S2 A:** Chromatogram of hair Cortisol & Internal standard (Cortisol D4).


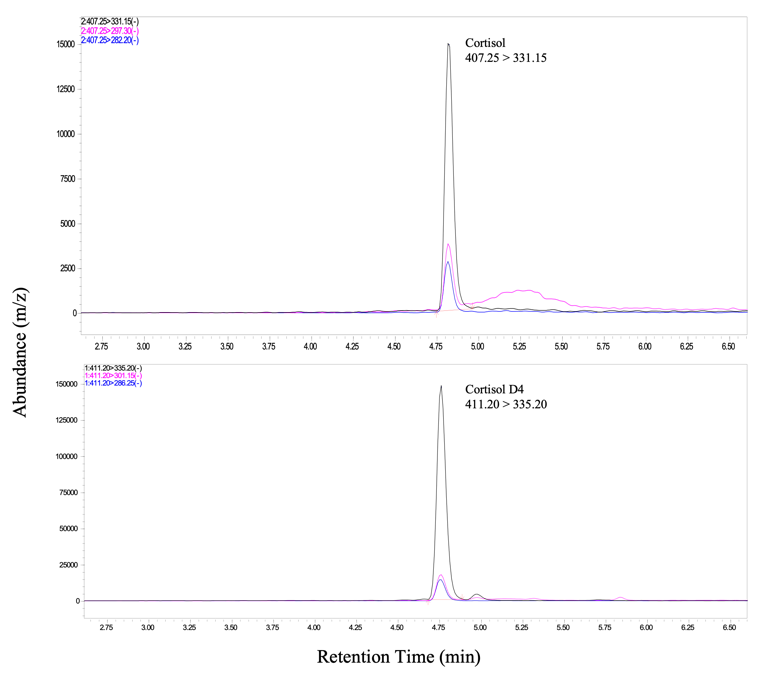


**Figure S2 B:** Chromatogram of saliva Cortisol & Internal standard (Cortisol D4).


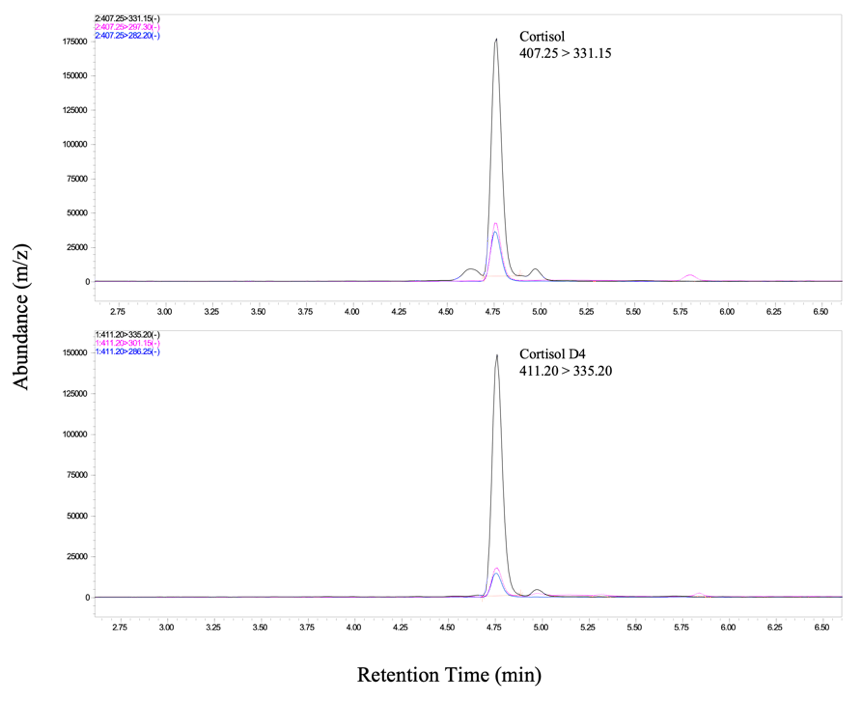


The distinctive elution behavior observed in the above chromatograms helps in the precise characterization and quantification of the targeted substances, validating the effectiveness of our methodology. By ensuring the validity of our research, this validation approach strengthens the validity of our study on student stress.

**Figure S3:** Hair cortisol levels among male (N=60) and female (N=37) students.

| 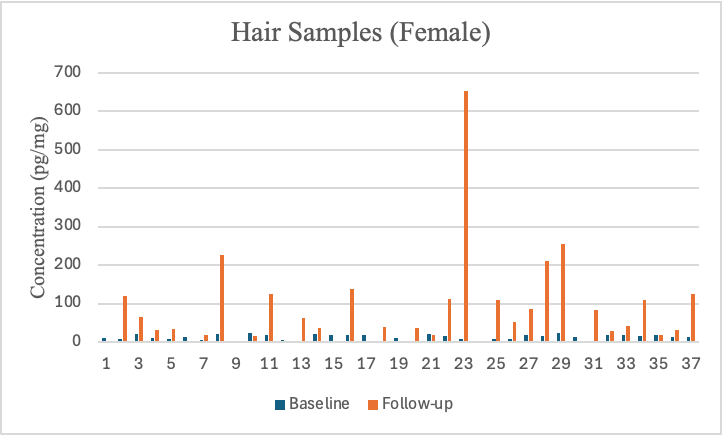 | 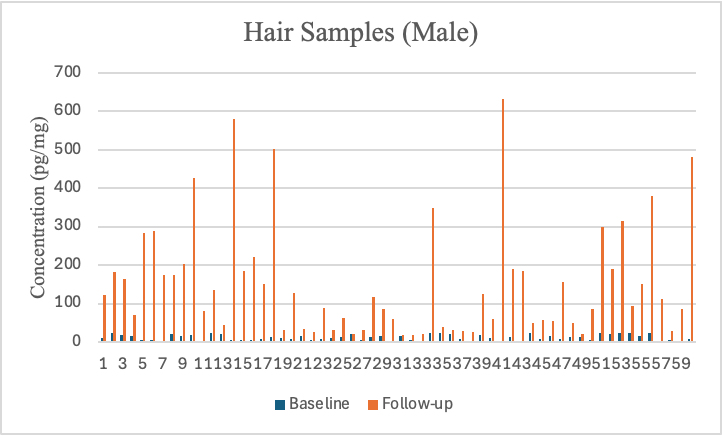 |
| --- | --- |

**Figure** **S4:** Saliva cortisol levels among male (N=60) and female (N=37) students

| 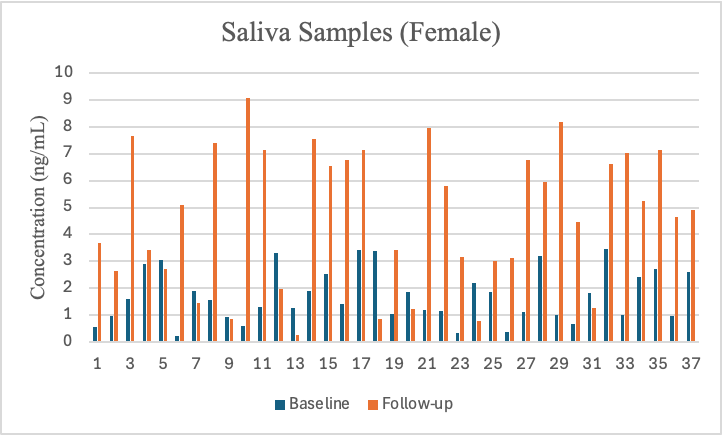 | 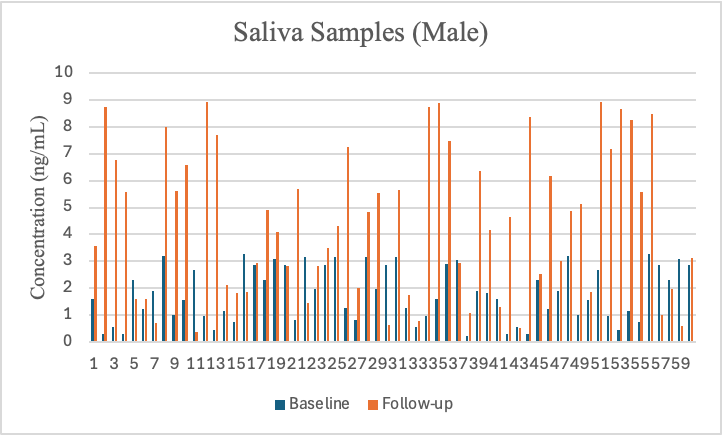 |
| --- | --- |

**Figure** **S5:** Estimation plot illustrating the differences in hair cortisol levels between baseline and follow-up samples for male (N=60) and female (N=37) students

**Figure** **S6:** Estimation plot showing the differences in salivary cortisol levels between baseline and follow-up samples for male (N=60) and female (N=37) students

**Figure** **S7:** Scatter plots showing the relationship between saliva and hair cortisol levels in students.
**(A)** Baseline samples show a weak and statistically non-significant correlation (r = 0.25, p > 0.05).


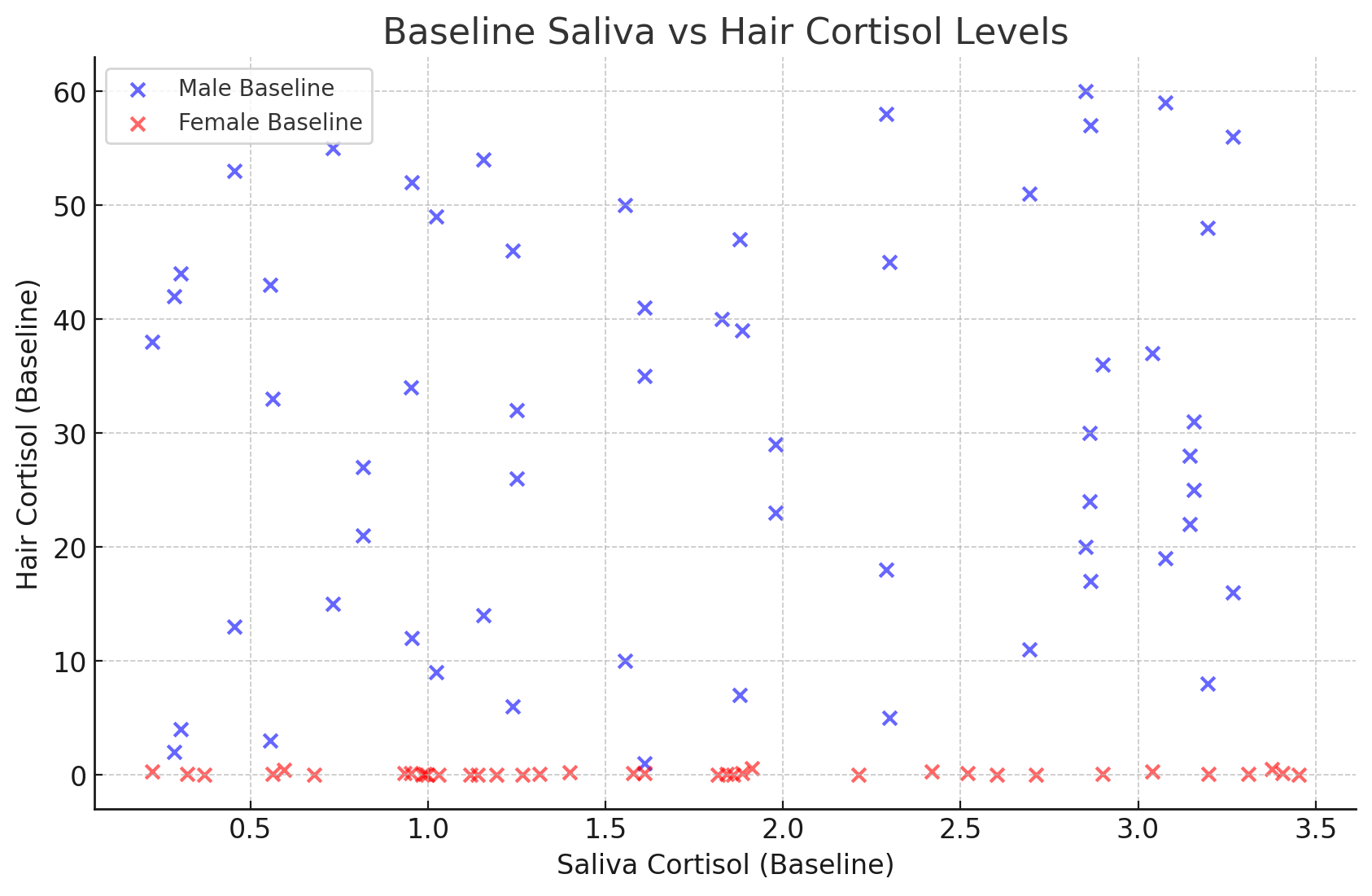


**(B)** Follow-up samples show a more substantial and statistically significant correlation (r = 0.68, p < 0.001).


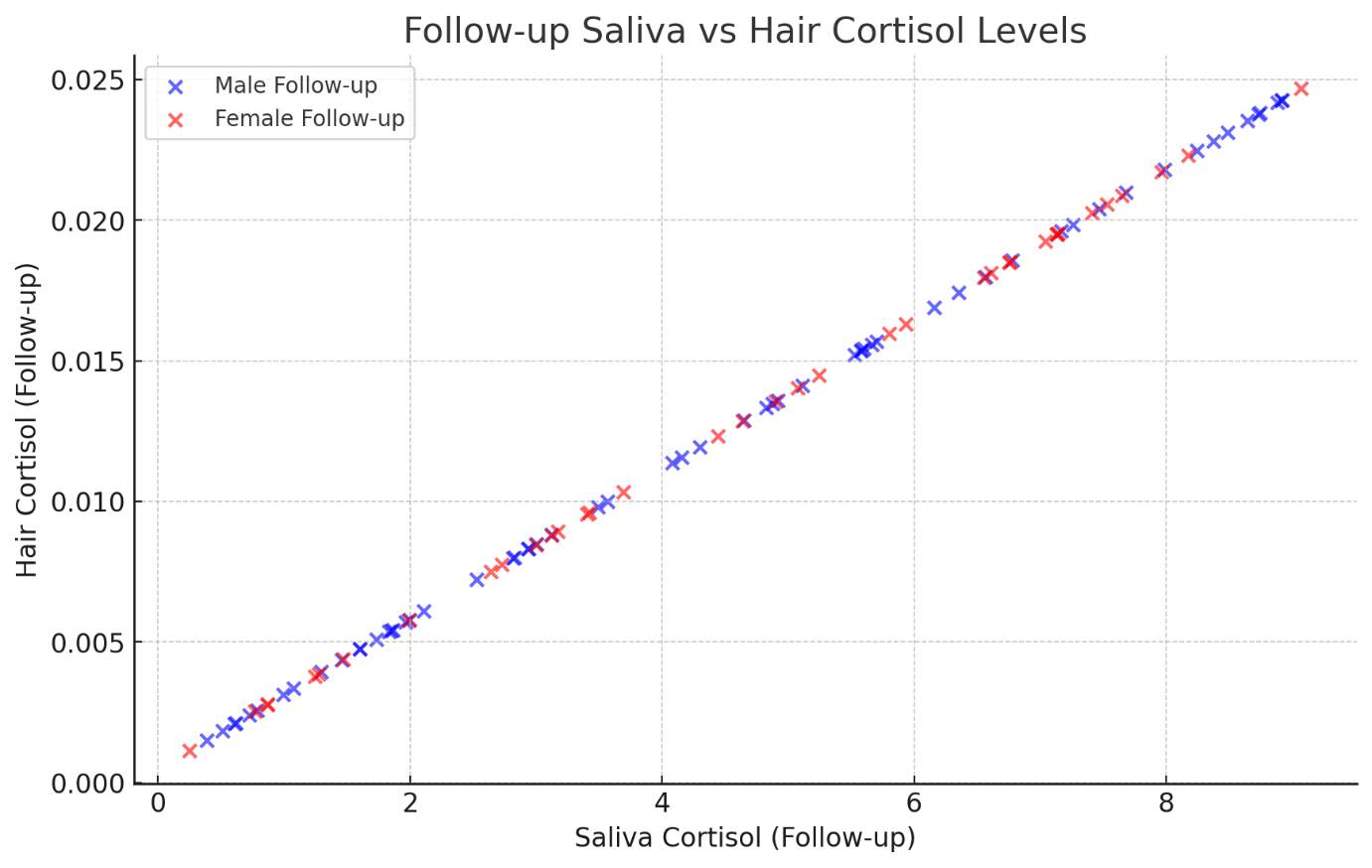

Supplement: Supplementary file 1 — Supporting Information. [file ANSA-6-e70003-s001.docx]
